# Liraglutide inhibits the apoptosis of human nucleus pulposus cells induced by high glucose through PI3K/Akt/caspase-3 signaling pathway

**DOI:** 10.1042/BSR20190109

**Published:** 2019-08-19

**Authors:** Yao Ming-yan, Zhang Jing, Guo Shu-qin, Bai Xiao-liang, Li Zhi-hong, Zhou Xue

**Affiliations:** 1 Department of Endocrinology, Baoding NO.1 Central Hospital, 320 Changcheng North Street, Lianchi District, Baoding City, Hebei Province, China; 2 Department of Cardiology, Affiliated Hospital of Hebei University, Baoding City, Hebei Province, China

**Keywords:** apoptosis, liraglutide, nucleus pulposus cells, oxidative stress, signaling pathway

## Abstract

Diabetes mellitus (DM) is a potential etiology of disc degeneration. Glucagon-like peptide-1 (GLP-1) is currently regarded as a powerful treatment option for type 2 diabetes. Apart from the beneficial effects on glycaemic control, GLP-1 has been reported to exert functions in a variety of tissues on modulation of cell proliferation, differentiation, and apoptosis. However, little is known regarding the effects of GLP-1 on nucleus pulposus cells (NPCs). In the present study, we investigated the effects of liraglutide (LIR), a long-lasting GLP-1 analogue, on apoptosis of human NPCs and the underlying mechanisms involved. We confirmed the presence of GLP-1 receptor (GLP-1R) in NPCs. Our data demonstrated that liraglutide inhibited the apoptosis of NPCs induced by high glucose (HG), as detected by Annexin V/Propidium Iodide (PI) and ELISA assays. Moreover, liraglutide down-regulated caspase-3 activity at intermediate concentration (100 nM) for maximum effect. Further analysis suggested that liraglutide suppressed reactive oxygen species (ROS) generation and stimulated the phosphorylation of Akt under HG condition. Pretreatment of cells with the Phosphoinositide 3-kinase (PI3K) inhibitor LY294002 (LY) and small interfering RNAs (siRNAs) GLP-1R abrogated the liraglutide-induced activation of Akt and the protective effects on NPCs’ apoptosis. In conclusion, liraglutide could directly protect NPCs against HG-induced apoptosis by inhibiting oxidative stress and activate the PI3K/Akt/caspase-3 signaling pathway via GLP-1R.

## Introduction and background

Intervertebral disc degeneration (IDD) is a common musculoskeletal disorder. It can induce spinal instability, stenosis, and neurothlipsis, which are the main causes of low back and leg pain [1]. Currently, the pathogenesis of IDD largely remains unclear. Moreover, we have not found an effective method to prevent the degeneration of intervertebral disc yet.

Recent studies have proved that diabetes mellitus (DM) is an important etiological factor of IDD [2,3]. Previous epidemiological studies have reported that the incidence of IDD in DM patients is higher than patients without DM, and the duration of DM was a risk factor for lumbar disc degeneration [4,5]. During IDD, nucleus pulposus cells (NPCs) apoptosis and accelerated ageing are considered as the two classical cellular processes [6,7]. Some studies have demonstrated that high glucose (HG) environment has adverse effects on the cell biology of intervertebral disc, such as inhibiting cellular proliferation and inducing cell apoptosis [8,9]. Hyperglycaemia can induce the excessive production of advanced glycolic end products and increase the formation of oxygen free radicals, which result in the mitochondria apoptosis through oxidative stress damage [10,11]. These findings suggest that inhibition of HG-induced aberrant apoptosis may be a potential strategy for delaying disc degeneration in diabetic patients.

Glucagon-like peptide-1 (GLP-1) is a key incretin hormone, secreted from enteroendocrine L cells to regulate glucose and energy homoeostasis [12]. GLP-1 exerts its effect mostly through the GLP-1 receptor (GLP-1R), which belongs to the G-protein coupled receptor family, in multiple tissues such as pancreas, heart, lung, stomach, intestine, kidney, brain, and bone [13–15]. Liraglutide (LIR), a long-lasting GLP-1 analogue with 97% homology with endogenous GLP-1, has been regarded as a powerful treatment option for type 2 diabetes. It exerts activities via the mediation of GLP-1R and effectively mimics the actions of mature GLP-1. At the level of cell kinetics, liraglutide could improve endothelial function, and influence various cellular pathways including anti-inflammatory and antioxidative stress [16,17]. However, to our knowledge, the role of GLP-1 in IDD has not been reported to date. Therefore, the present study was conducted to investigate the effects of GLP-1 analogue, liraglutide, on the HG-induced apoptosis of NPCs and the underlying molecular mechanisms involved.

## Materials and methods

### Cell culture and experimental design

Human NPCs were purchased from American Science Cell Research Laboratories and cultured in NPC Medium (NPCM) containing 500 ml basal medium, 10 ml Foetal Bovine Serum (FBS), 5 ml NPC Growth Supplement, and 5 ml penicillin/streptomycin solution (P/S) (HyClone, Logan, UT, U.S.A.) at 37°C, 5% CO_2_ in a humidified incubator. The culture medium was refreshed the next day to remove unattached cells and residual DMSO (Solarbio, Beijing, China), and then it was changed every 2–3 days. When NPCs reached approximately 80–90% confluence, they were split and subcultured at a 1:3 ratio with a 0.25% (w/v) trypsin (Sigma, St.Louis, MO, U.S.A.) solution. The third-generation NPCs were randomly divided as follows: (1) Control (CON) group: cultured in NPCM; (2) HG group: cultured in 0.2 M HG concentration; (3) HG+LIR group: cultured in HG medium containing various concentrations of liraglutide (10, 100, or 1000 nM); (4) HG+LIR+LY group: cultured in HG medium containing liraglutide and LY294002 (LY, 20 μM). The cellular morphology was observed by inverted phase-contrast microscopy (Olympus, Tokyo, Japan). The cell vitality, apoptosis rate, reactive oxygen species (ROS) level, and the expression of GLP-1R, Akt, p-Akt, and caspase-3 of the groups were detected under the same experimental conditions after incubation for 48 h.

### Cell viability assay

Cell viability was tested using EdU (5-ethynyl-2′-deoxyuridine) assay kit (Sigma–Aldrich, U.S.A.) according to its protocol. Briefly, the cells (density 1.0 × 10^5^/ml) were plated in 96-well plates (three wells for each group and 100 μl in each well) in a CO_2_ incubator, and 10 μM EdU was added at same time. After incubation for 4, 8, 16, 24, 36, and 48 h, respectively, cells were analysed using a TCS SP8 confocal microscope (Leica Microsystems, Germany). DNA (blue) was stained with Hoechst 33342 (ab145597, Abcam, U.S.A.). Green cells show EdU/Hoechst-positive cells.

### Detection of apoptosis by flow cytometric analysis

Cell apoptosis was assessed by an Annexin V-FITC/Propidium Iodide (PI) apoptosis detection kit (BD Pharmingen, U.S.A.). Briefly, NPCs were trypsinised with 0.25% trypsin (Sigma, St. Louis, MO, U.S.A.) and washed twice with phosphate buffer solution (PBS, Solarbio, China) after centrifugation at 4°C. The cells were resuspended in 200 μl of binding buffer and mixed with 10 μl of Annexin V-FITC solution in the dark at room temperature for 15 min. Then 10 μl of PI and 300 μl of binding buffer were added for another 5 min. Thereafter, NPCs were subjected to a flow cytometry machine (BD Biosciences, San Jose, CA, U.S.A.) to analyse the apoptosis ratio. Apoptotic cells, stained positive for Annexin V-FITC, negative for PI, and double positive, were counted. Data were represented as a percentage of the total cell count.

### Apoptosis determination by ELISA

Cell death detection ELISA plus kit (Roche Molecular Biochemicals, Germany) was used to measure cell apoptosis according to manufacturer’s instructions. NPCs were exposed to HG and various concentrations of liraglutide for 48 h, and then lysed for 30 min at room temperature, followed by centrifugation for 10 min. DNA fragments detected in the supernatants showed the extent of apoptosis in the sample.

### Caspase-3 activity assay

Caspase-3 activity was determined using a commercial caspase-3 activity kit (Beyotime, China). As recommended by the manufacturer’s protocol, treated NPCs were washed with cold PBS for three times and lysed with lysis buffer (100 μl per 2 × 10^6^ cells) for 15 min at 4°C. The protein supernatant was obtained by centrifugation for 15 min. After incubating the reaction mixture system composed of 10 μl of cell lysate, 80 μl of detection buffer solution, and 10 μl of Ac-DEVD-pNA (2mM) in 96-well microtitre plates at 37°C for 4 h, caspase-3 activity was quantified by detecting the absorbance value at an absorbance of 405 nm with a microplate spectrophotometer (Biotek, Winooski, VT, U.S.A.). Caspase-3 activity was expressed as the fold change in enzyme activity over control.

### Detection of intracellular ROS

#### ROS levels by flow cytometric analysis

NPCs were treated according to the experiment grouping design, and 10 μM resveratrol was used as the positive control. After culturing the cells with HG and 0.5% FBS DMEM for 48 h, the intracellular production of ROS in each group was detected by loading the cultured cells with the fluoroprobe carboxymethyl-H2-dichlorofluorescein diacetate (1 μM) (CM-H2-DCF-DA, Sigma Chemical Co., St. Louis, MO, U.S.A.), a nonpolar compound that is converted into a nonfluorescent polar derivative 2′,7′-dichlorofluorescin (DCFH) by cellular esterase after incorporation into cells. Fluorescence intensity was determined immediately with a flow cytometer (excitation λ = 488 nm, emission λ = 515 nm, BD FACSCalibur system, Franklin Lakes, NJ). The average fluorescence intensity was normalised by the total cell protein content of each experimental group of cells as previously described [18].

### Western blotting analysis

Protein levels of GLP-1R, Akt, p-Akt (T308), and cleaved-caspase-3 were detected by Western blot, with GAPDH as internal reference protein following previous studies [19,20]. NPCs were lysed using the RIPA lysis buffer (Beyotime, China) on the ice for 20 min and the protein concentration was quantified with a Protein BCA Kit (Beyotime, China). Equal amounts of protein samples were subjected to 10% sodium dodecyl sulfate polyacrylamide gel electrophoresis and transferred to the PVDF membrane (Merck Millipore, Billerica, MA, U.S.A.). The membranes were blocked with 5% non-fat milk in TBST (50 mmol/l Tris, pH 7.6, 150 mmol/l NaCl, 0.1%) for 1 h at the room temperature and incubated with specific primary antibodies (Proteintech, Wuhan, China) at 4°C overnight. After washing three times in TBST, membranes were incubated with the secondary antibodies (Proteintech, Wuhan, China) for 2 h at 37°C. The enhanced chemiluminescence (ECL) reagent (Thermo, U.S.A.) was used to detecte protein bands, and grey values were analysed using ImageJ software (National Institutes of Health, U.S.A.).

### Immunofluorescence

NPCs were cultured on a 15-mm confocal dish (catalogue no. 801002, NEST). Cells were washed three times with PBS, fixed in 4% formaldehyde for 15 min at room temperature, and blocked with 3% bovine serum albumin/0.3% Triton X-100 in PBS. The cells were subsequently incubated with anti-GLP-1R antibody overnight at 4°C. Finally, the cells were incubated with Alexa Fluor VR 488 donkey anti-rabbit IgG (A11008, Life Technologies, Waltham, MA, U.S.A.) for 2 h and contained with DAPI (4,6-diamidino-2-phenylindole). The images of stained cells were obtained using a multiphoton laser scanning microscope (FV1000 MPE, Olympus, Tokyo, Japan) [21].

### Quantitative real-time PCR

Briefly, total RNA was extracted using the TRIzol reagent (Invitrogen, U.S.A.) and quantified fluorometrically using a CyQuant-Cell Proliferation Assay Kit (Molecular Probes, Eugene, OR, U.S.A.), and then reverse-transcribed into cDNA using a ThermoScript RT Kit (Invitrogen, Shanghai, China) according to the recommended protocol. Primers designed by Primer Premier Version 5.0 software were used to amplify the target genes (Table 1). To investigate the expression of mRNA, cDNA was amplified using a GoScript™ Reverse Transcription System (Promega, U.S.A.) in a standard 20-μl reaction volume. The PCR parameters consisted of an initial denaturation for 3 min at 95°C and 40 cycles of two steps (95°C for 10 s, and 60°C for 30 s). Standard curves were run in each assay that produced a linear plot of threshold cycle (*C*_t_) against log (dilution). Target gene expression was quantified based on the concentration of the standard curve. Data were presented as relative *C*_t_ values (*n*=3). Relative gene expression data were normalised to the reference gene (*GAPDH*) according to the 2^−ΔΔ*C*^_t_ method.

**Table 1:** Real-time PCR primers

Gene	Primer sequences
*Caspase-3*	F: 5′-TGTTTCCCTGAGGTTTGCTG-3′
	R: 5′-TGCTATTGTGAGGCGGTTGT-3′
*GAPDH*	F: 5′-AACTTTGGCATCGTGGAAGGG-3′
	R: 5′-AGGGATGATGTTCTGGGCTGC-3′

Sequences of forward (F) and reverse (R) primers are shown.

### Small interfering RNA

GLP-1R expression in NPCs was silenced by transfection of small interfering RNA (siRNA). NPCs were transfected with GLP-1R siRNA (GenePharma Biotechnology, Shanghai, China) using Entranster™-R4000 (Engreen Biosystem Co, Ltd.) according to the instructions. The siRNA silencing efficiency was determined 48 h post transfection by protein analysis for further experiments.

### Statistical analysis

Statistical analyses were performed by SPSS 22.0 software. All data were expressed as mean ± standard deviation (SD) from the results of at least three independent experiments. Statistical analysis among multiple groups was analysed using the one-way analysis of variance (ANOVA), and the post hoc test was performed using the SNK-q test or LSD test. *P*<0.05 was considered statistically significant.

## Results

### GLP-1R expression in NPCs

**Figure 1:**
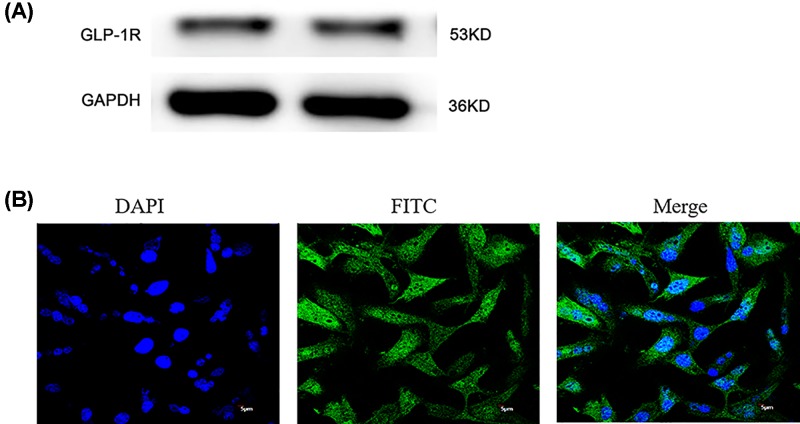
GLP-1R was expressed in NPCs by Western blot and immunofluorescence (**A**) Western blot indicated the presence of the GLP-1R in NPCs. (**B**) Immunofluorescence confirmed that GLP-1R was located at the surface of NPCs.

To elucidate the role of GLP-1 in NPCs apoptosis, we demonstrated the expression of GLP-1R protein in NPCs with Western blot analysis (Figure 1).

### LIR increased the proliferation activity in HG-induced NPCs

**Figure 2:**
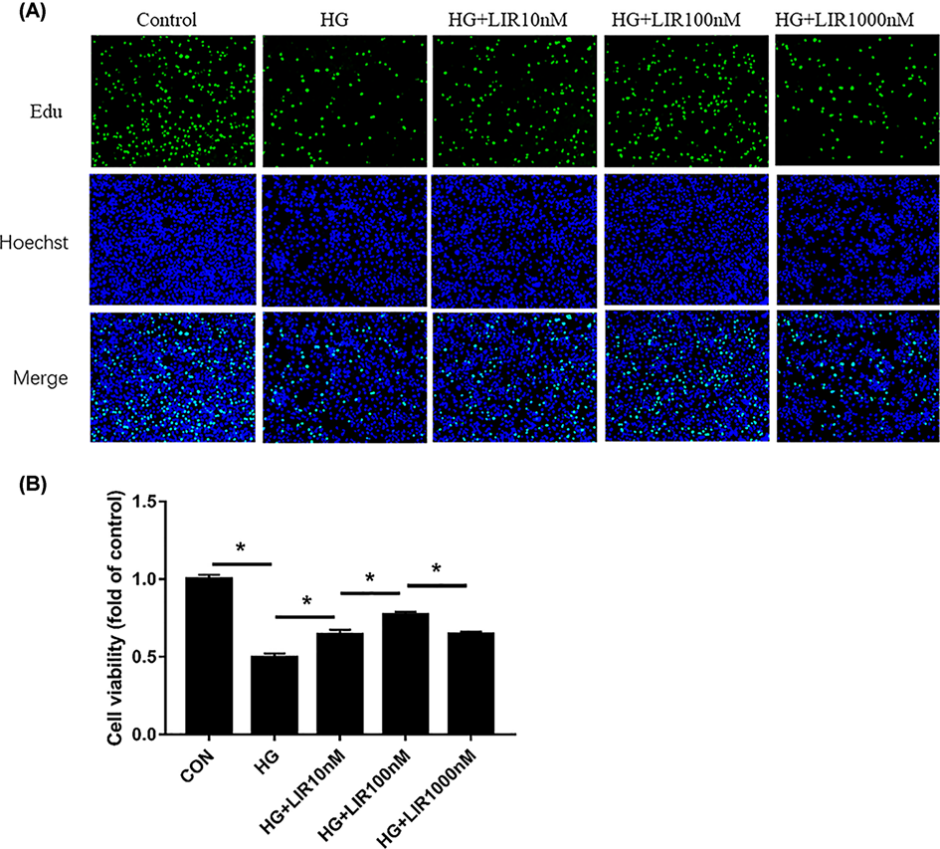
Effect of liraglutide on HG-induced proliferation activity in NPCs by EdU assay (**A**) Representative figures of EdU assay to examine cell activity in NPCs. (**B**) Quantitative analysis of cell viability by comparing with normal cultured control. Data are presented as mean ± SD (*n*=3). *P<0.05.

To ascertain the role of liraglutide in cell proliferation, we examined the viability of NPCs incubated with HG and different doses of liraglutide for 48 h with six time points using EdU assay. The results of EdU assay showed that the cell proliferation activity in the HG group significantly decreased compared with the control group (*P*<0.05). The HG in addition to different doses of liraglutide groups showed increased cell proliferation activity compared with the HG group (*P*<0.05). And we noted that liraglutide concentrations up to 10 nM showed significant protective effect on HG treated NPCs, and the maximal increase in proliferation activity was at 100 nM liraglutide. The differences among different groups were statistically significant (Figure 2).

### Liraglutide inhibited HG-induced apoptosis in NPCs

**Figure 3:**
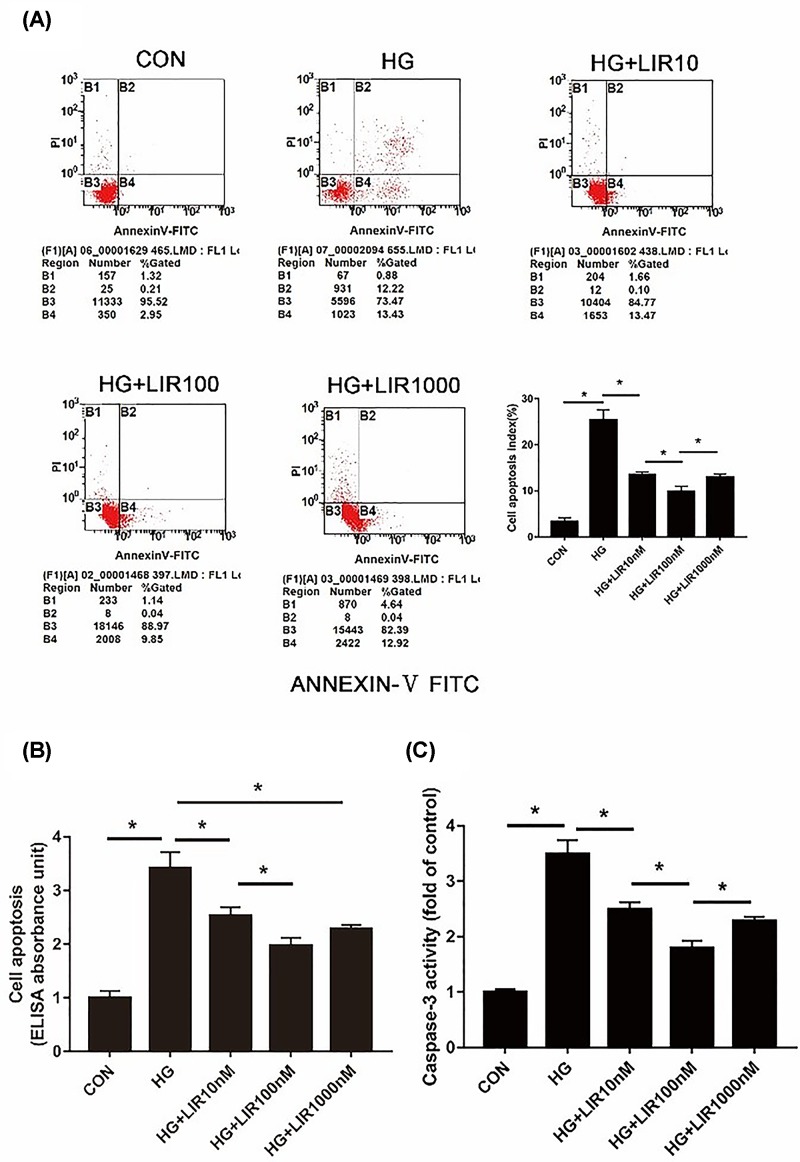
Effect of liraglutide on HG-induced apoptosis in NPCs Cells were exposed to HG and liraglutide (0, 10, 100, or 1000 nM) for 48 h. (**A**) Apoptosis was determined by Annexin V-PI double staining. The percentage of apoptotic cells by flow cytometry was higher for cells treated with HG than control group, which could be effectively decreased by the use of liraglutide. (**B**) Cell death detection by ELISA showed that HG increased the apoptosis of NPCls and liraglutide suppressed it. (**C**) The caspase-3 activity was measured using a commercial caspase-3 activity kit (Beyotime, China). Caspase-3 activity is expressed as the fold change in enzyme activity over control. Data are presented as mean ± SD (*n*=3). *P<0.05.

Apoptotic NPCs were detected by both Annexin V/PI staining and Cell Death ELISA. As shown in Figure 3A,B, HG (0.2 M) resulted in a marked increase in apoptotic incidence, which could be effectively decreased by the use of liraglutide. Meanwhile, caspase-3 activity, a crucial mediator of cell apoptosis, was significantly increased after treating with HG compared with the control group, and it was significantly decreased after liraglutide treatment (*P*<0.05) (Figure 3C). Additionally, apoptosis analysis showed that liraglutide exerted the maximal anti-apoptotic effect at concentration of 100 nM, which was selected as the optimal concentration for subsequent experiments.

### Liraglutide decreased HG-induced ROS generation in NPCs

**Figure 4:**
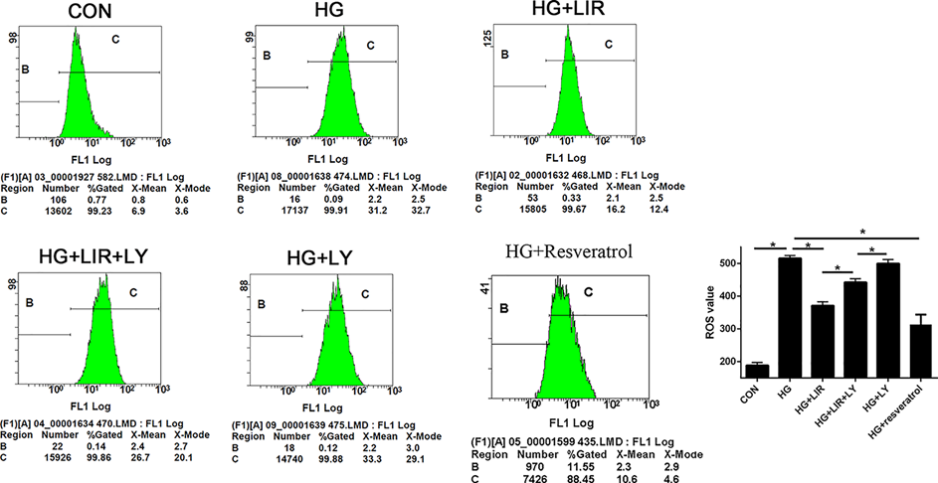
Effect of liraglutide on HG-induced ROS generation in NPCs Intracellular ROS rates were determined by flow cytometric analysis. Liraglutide attenuated the HG-induced ROS generation, to a certain extent. When PI3K/Akt pathway was blocked by LY, the inhibition of liraglutide was weakened. A total of 10 μM resveratrol was used as positive control. Data are presented as mean ± SD (*n*=3). *P<0.05. Abbreviation: PI3K, phosphoinositide 3-kinase .

The results of ROS detection in each group are shown in Figure 4. We observed that HG induced the increase in intracellular ROS rate, and the addition of liraglutide obviously decreased it (*P*<0.05). Meanwhile, the inhibitor LY partly reversed the effect of liraglutide in the HG group (*P*<0.05), whereas the inhibitor LY added alone in HG did not significantly affect the generation of ROS compared with HG group (*P*>0.05).

### Liraglutide inhibited HG-induced NPCs apoptosis via phosphoinositide 3-kinase/Akt/caspase-3 signaling pathway

**Figure 5:**
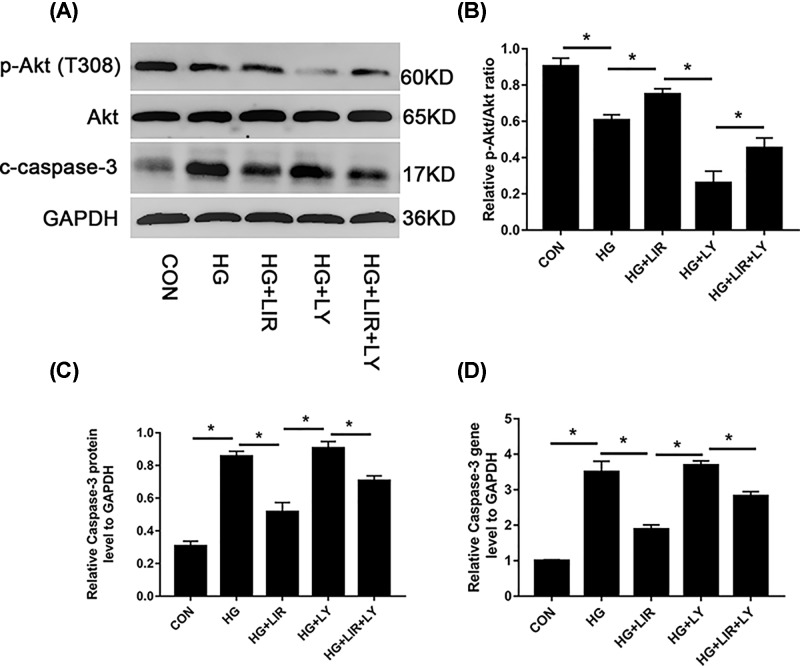
Activation of PI3K/Akt/caspase-3 signaling pathway in liraglutide-stimulated NPCs Cells were exposed to 0.2 M HG, 100 nM liraglutide, and 20 μM LY, respectively. (**A**) Representative Western blot picture. (**B**) Quantitation analysis of ratio of p-Akt/total AKT. (**C**) Quantitation of cleaved caspase-3 expression standardised by GAPDH. (**D**) Caspase mRNA expression among all groups determined by QRT-PCR. Data are presented as mean ± SD (*n*=3). *P<0.05.

To investigate the molecular mechanism by which liraglutide exerts the effects of anti-apoptosis, the activation of intracellular signalling pathways was examined. Results showed that gene expression of caspase-3 was up-regulated by HG (*P*<0.05). The addition of liraglutide decreased mRNA levels of caspase-3, which was partly reversed by the inhibitor LY (*P*<0.05) (Figure 5D). The protein expression of caspase-3, Akt, and p-Akt were detected by Western blot assay. Our data showed that 100 nM liraglutide induced the phosphorylation of Akt, which was blocked by the phosphoinositide 3-kinase (PI3K) inhibitor LY (*P*<0.05). Besides, the expression of caspase-3 was up-regulated by HG, but then down-regulated by the addition of liraglutide (*P*<0.05). The inhibitor LY could partly increase the expression of caspase-3 when compared with liraglutide group (*P*<0.05) (Figure 5A–C).

**Figure 6:**
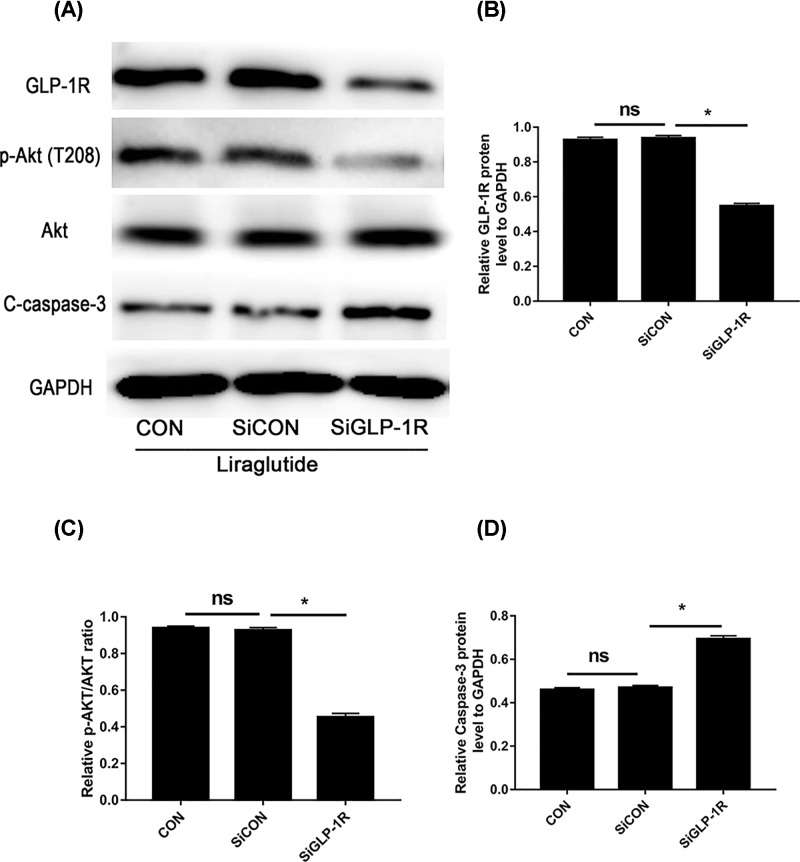
GLP-1R knockdown significantly abolished liraglutide-induced activation of PI3K/Akt signaling in NPCs NPCs were transfected with negative control siRNA (SiCON) or GLP-1R siRNA (SiGLP-1R) before liraglutide treatment. (**A**) Representative Western blot picture. (**B**) Quantitation of GLP-1R expression standardised by GAPDH. (**C**) Quantitation analysis of ratio of p-Akt/total AKT. (**D**) Quantitation of cleaved caspase-3 expression standardised by GAPDH. Data are presented as mean ± SD (*n*=3). *P<0.05. ns: non-significant.

In addition, suppression of GLP-1R with siRNA blocked the phosphorylation of Akt and increased the expression of caspase-3 after liraglutide treatment, when compared with both the control and negative control siRNA group (Figure 6A–D). These results indicate that liraglutide stimulates the PI3K/Akt/caspase-3 signal transduction pathway via GLP-1R in NPCs.

## Discussions

The current study explored the effects of liraglutide on the apoptosis of NPCs induced by HG. Our findings supported the protective role that liraglutide exerted on HG-induced damage in NPCs. This effect was mediated, at least in part, through inhibition of oxidative stress and activation of PI3K/Akt/caspase-3 pathway.

Disc degeneration is a common disease which brings enormous socioeconomic burden to healthcare system worldwide [22]. Recent basic and epidemiological studies have shown that DM is an important etiological factor of disc degeneration [4,23]. Previously, some studies demonstrated that HG could cause negative effects on disc cell’s biology, such as inducing disc cell apoptosis and senescence [24,25], and oxidative injury was a vital mechanism participating in the detrimental effects [26]. In our study, the HG group exhibited a significant increase in the formation of ROS levels and higher apoptosis rate of NPCs compared with the control group, which were consistent with previous findings. Oxidative stress arises from the imbalance between the generation of oxygen-derived ROS and antioxidation defence when the body is exposed to harmful stimuli. In a high-glucose environment, cells produce excessive intracellular ROS, which can induce peroxidation of lipid, leading to a loss of membrane integrity, the depolarisation of mitochondrial membrane, and the activation of apoptotic signaling pathways, including the overexpression of caspase 9/3 [27,28]. The activation of mitochondrion-mediated apoptosis pathway results in cell apoptosis eventually. Increased apoptosis is a significant risk factor for IDD [6]. Therefore, the inhibition of HG-mediated oxidative damage and apoptosis may be a potential strategy to retard disc degeneration.

Beyond the beneficial effects on glycaemic control, GLP-1 has been reported to play a variety of functions in different tissues on regulation of cell proliferation, differentiation, and apoptosis [29]. Of interest, it has been shown as an anti-apoptotic factor in various cells, such as cardiomyocytes [30], pancreatic β-cells [31], and MC3T3-E1 cells [32]. Nevertheless, little is known about the role of GLP-1 in IDD. Liraglutide, a long-lasting analogue of GLP-1, exerts its physiological role by binding to GLP-1R, and our results ascertained that GLP-1R was expressed on NPCs, which has not been reported up to now. In our study, we found that liraglutide could partly alleviate HG (0.2 M) induced NPCs apoptosis and intracellular ROS generation, and the maximum effect achieved at an intermediate concentration (100 nM), not in a dose-dependent manner. One possible reason for this phenomenon is that a minor concentration of liraglutide may not cause significant anti-apoptotic effect, while a higher stimulation may have negative effects. This is because GLP-1Rs belong to GPCR, and some desensitisation or rapid attenuation of receptor sensitivity may occur after exposure to agonists [33].

Moreover, our study found that liraglutide attenuated the activity of caspase-3, which is a critical enzyme for cell survival and apoptosis [34]. Multiple mechanisms may be related to the anti-apoptotic effects of liraglutide in NPCs. The PI3K/Akt pathway plays an important role in regulating cell proliferation, senescence, and apoptosis [35]. It has been reported to participate in NPCs apoptosis in previous studies [36]. To our knowledge, liraglutide can activate PI3K/Akt signaling cascade [16,32]. Our data showed that liraglutide partly up-regulated the level of p-Akt/Akt and decreased the expression of caspase-3 in HG environment. The activity of the PI3K/Akt pathway and ROS generation showed contrary changes in the liraglutide-treated group. Furthermore, the activation of PI3K/Akt pathway was blocked by the inhibition of GLP-1R with siRNA, which suggests that the downstream signaling pathway is mediated through GLP-1R. Both suppression of Akt and silence of GLP-1R offset the protective effects of liraglutide on HG-induced NPCs apoptosis, indicating that liraglutide prevents NPCs from apoptosis by activating the PI3K/Akt/caspase-3 signaling pathway through GLP-1R.

Liraglutide antioxidation effect was also partially explained by previous researches. Several studies reported that administration of GLP-1 could suppress glucose-stimulated inducible nitric oxide synthase activity and expression and its stimulation of insulin release in pancreatic islet cells, reduced the level of derivatives of reactive oxygen metabolites [37]. Liraglutide also inhibited rapid translocation of PKC-α into membrane, inhibited NF-κB signaling activation and NADPH oxidase, increased the levels of SOD-2, catalase and GPx [38].

In conclusion, the present study suggested that liraglutide could attenuate HG-induced NPCs apoptosis, and the inhibition of oxidative stress and activation of PI3K/Akt/caspase-3 pathway may be responsible for its protective effects. Therefore, liraglutide as an antidiabetic agent may confer a beneficial effect in disc degeneration through regulation of NPCs apoptosis, and may be a potential therapeutic strategy for retarding disc degeneration in DM patients, which still requires further clarification.

## Abbreviations

*C*_t_: threshold cycle
DM: diabetes mellitus
EdU: 5-ethynyl-2′-deoxyuridine
FBS: foetal bovine serum
GLP-1: glucagon-like peptide-1
GLP-1R: GLP-1 receptor
HG: high glucose
IDD: intervertebral disc degeneration
LIR: liraglutide
LY: LY294002
NPC: nucleus pulposus cell
NPCM: NPC medium
PBS: phosphate buffer solution
PI: propidium iodide
PI3K: phosphoinositide 3-kinase
ROS: reactive oxygen species
siRNA: small interfering RNA
